# Epidemiologic and Clinical Features of Mpox-Associated Deaths — United States, May 10, 2022–March 7, 2023

**DOI:** 10.15585/mmwr.mm7215a5

**Published:** 2023-04-14

**Authors:** Aspen P. Riser, Allison Hanley, Michael Cima, Linda Lewis, Kayla Saadeh, Jemma Alarcón, Lauren Finn, Moon Kim, Jeremy Adams, Douglas Holt, Amanda Feldpausch, Jessica Pavlick, Andrew English, Marguerite Smith, Tyler Rehman, Ronald Lubelchek, Stephanie Black, Matthew Collins, Layne Mounsey, David Blythe, Meredith Hodach Avalos, Ellen H. Lee, Olivia Samson, Marcia Wong, B. Denise Stokich, Ellen Salehi, Lynn Denny, Kirsten Waller, Pamela Talley, Julie Schuman, Michael Fischer, Stephen White, Kenneth Davis, Ashley Caeser Cuyler, Rabeeya Sabzwari, Robert N. Anderson, Katrina Byrd, Jeremy A. W. Gold, Shannon Kindilien, James T. Lee, Siobhán O’Connor, Jesse O’Shea, LaTweika A. T. Salmon-Trejo, Raquel Velazquez-Kronen, Carla Zelaya, William Bower, Sascha Ellington, Adi V. Gundlapalli, Andrea M. McCollum, Leah Zilversmit Pao, Agam K. Rao, Karen K. Wong, Sarah Anne J. Guagliardo

**Affiliations:** ^1^Mpox Emergency Response Team, CDC; ^2^Epidemic Intelligence Service, CDC; ^3^Arkansas Department of Health; ^4^California Department of Health; ^5^Los Angeles County Department of Public Health, Los Angeles, California; ^6^Florida Department of Health; ^7^Georgia Department of Public Health; ^8^Illinois Department of Public Health; ^9^Department of Infectious Diseases, Loyola University Chicago, Chicago, Illinois; ^10^Cook County Department of Public Health, Forest Park, Illinois; ^11^Chicago Department of Public Health, Chicago, Illinois; ^12^Indiana Department of Health; ^13^Maryland Department of Health; ^14^New Jersey Department of Health; ^15^New York City Department of Health and Mental Hygiene, New York, New York; ^16^Nevada Department of Health and Human Services; ^17^Ohio Department of Health; ^18^Pennsylvania Department of Health; ^19^Tennessee Department of Health; ^20^Texas Department of State Health Services; ^21^Virginia Department of Health; ^22^Edward Hines Veterans Affairs Medical Center, Hines, Illinois.

As of March 7, 2023, a total of 30,235 confirmed and probable monkeypox (mpox) cases were reported in the United States,^†^ predominantly among cisgender men^§^ who reported recent sexual contact with another man (*1*). Although most mpox cases during the current outbreak have been self-limited, cases of severe illness and death have been reported (*2**–**4*). During May 10, 2022–March 7, 2023, 38 deaths among persons with probable or confirmed mpox^¶^ (1.3 per 1,000 mpox cases) were reported to CDC and classified as mpox-associated (i.e., mpox was listed as a contributing or causal factor). Among the 38 mpox-associated deaths, 94.7% occurred in cisgender men (median age = 34 years); 86.8% occurred in non-Hispanic Black or African American (Black) persons. The median interval from symptom onset to death was 68 days (IQR = 50–86 days). Among 33 decedents with available information, 93.9% were immunocompromised because of HIV. Public health actions to prevent mpox deaths include integrated testing, diagnosis, and early treatment for mpox and HIV, and ensuring equitable access to both mpox and HIV prevention and treatment, such as antiretroviral therapy (ART) (*5*). 

Data included in this report were collected during May 10, 2022–March 7, 2023. Jurisdictional health departments electronically reported confirmed and probable mpox cases and associated deaths as part of national case surveillance. Case data were shared with CDC through a standardized case report form or through the National Notifiable Diseases Surveillance System.** Additional data (e.g., clinical course, co-occurring health conditions, and treatments received) about some decedents were collected during consultations between treating clinicians and CDC clinical officers.^††^ Cause of death was most commonly determined by the treating health care provider and reported on the death certificate. Jurisdictional health departments shared cause-of-death data as reported on the death certificate to support classification of deaths. Deaths were classified as mpox-associated if mpox was listed on Part I or Part II of the death certificate (chain of events that directly caused the death or significant conditions contributing to death, respectively). Deaths were classified as non–mpox-associated if mpox was not listed on the death certificate, or if mpox appeared to be incidental to death. Deaths were classified as being under investigation^§§^ if jurisdictions were reviewing the cause-of-death with health care providers at the time of this analysis.

Descriptive statistics on demographics (e.g., age, gender identity, race and ethnicity, and state of residence) and clinical characteristics (i.e., treatments received and timing of treatments) were calculated for all patients with an mpox-associated death and were compared between decedents and patients who are presumed to have survived after confirmed or probable mpox (survivors). SAS statistical software (version 9.4; SAS Institute) was used for all analyses. This activity was reviewed by CDC and was conducted consistent with applicable federal law and CDC policy.^¶¶^

CDC received reports of 52 deaths among persons with confirmed or probable mpox. Thirty-eight deaths (73.1%) were classified as mpox-associated, three (5.8%) were non–mpox-associated,*** and 11 (21.2%) deaths remain under investigation. Among the 38 mpox-associated deaths, 25 (65.8%) occurred during October–November 2022 (Figure).

**FIGURE:**
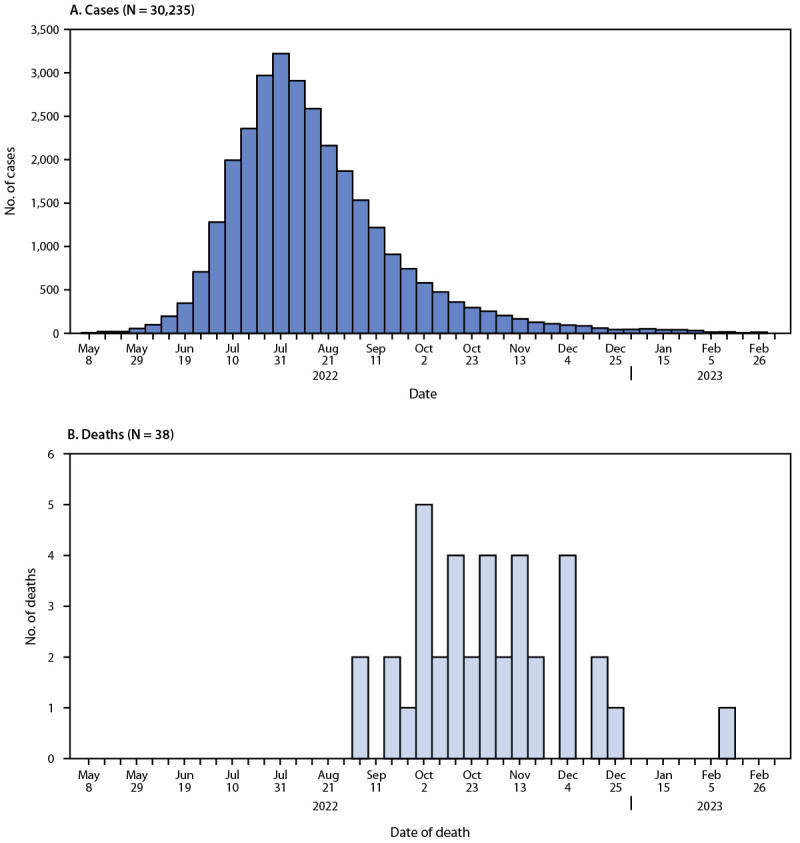
Weekly confirmed and probable* mpox cases (A)^†^ and mpox-associated deaths (B),^§^ by week — United States, May 10, 2022—March 7, 2023 * https://www.cdc.gov/poxvirus/mpox/clinicians/case-definition.html ^†^ The earliest date available regarding the case, beginning with date of illness or rash onset, diagnosis date, positive laboratory test report date, CDC call center reporting date, or case data entry date into the National Notifiable Diseases Surveillance System. ^§^ Date of death as reported on the death certificate.

The median age of decedents was 34 years (range = 22–58 years), and 36 (94.7%) were cisgender men (Table 1). A higher proportion of decedents than survivors were Black (86.8% versus 32.9%). Nearly one half of decedents (47.4%) resided in the U.S. Census Bureau South Region^†††^ compared with 39.4% of survivors who lived in this region. Nine of the 10 decedents with recent sexual or intimate contact information had sexual or intimate contact with cisgender men in the 3 weeks preceding symptom onset. Two decedents reported nonsexual close contact exposures (e.g., co-sleeping and caring for a household member) to persons with mpox. Five of 11 decedents with available data were experiencing homelessness. Among 87% of decedents and 45% of survivors with available information, HIV infection was more prevalent among decedents than survivors (93.9% versus 38.3%).

**TABLE 1:** Demographic and epidemiologic characteristics of persons who survived or died* from mpox-related illness — United States, May 10, 2022–March 7, 2023

Characteristic	Mpox cases, no. (%)^†^	
	Survivors (n = 30,183)	Decedents (n = 38)
**Demographic**		
**Median age, yrs (range)**	**34 (0–89)**	**34 (22–58)**
**Sex or gender,^§^ total**	**26,082 (86.4)**	**38 (100.0)**
Cisgender man	24,759 (94.9)	36 (94.7)
Cisgender woman	806 (3.1)	1 (2.6)
Transgender man	55 (0.2)	0 (—)
Transgender woman	227 (0.9)	1 (2.6)
Another gender identity	235 (0.9)	0 (—)
Unknown	4,101	0
**Race and ethnicity, total**	**28,233 (93.5)**	**38 (100.0)**
American Indian or Alaska Native, non-Hispanic	115 (0.4)	0 (—)
Asian, non-Hispanic	786 (2.8)	0 (—)
Black or African American, non-Hispanic	9,295 (32.9)	33 (86.8)
Native Hawaiian or other Pacific Islander, non-Hispanic	68 (0.2)	0 (—)
White, non-Hispanic	8,277 (29.3)	3 (7.9)
Hispanic or Latino	8,849 (31.3)	2 (5.3)
Other race, non-Hispanic	668 (2.4)	0 (—)
Multiple races, non-Hispanic	175 (0.6)	0 (—)
Unknown	1,950	0
**U.S. Census Bureau region,^¶^** **total**	**30,183 (100.0)**	**38 (100.0)**
Northeast	6,600 (21.9)	6 (15.8)
Midwest	3,164 (10.5)	9 (23.7)
South	11,882 (39.4)	18 (47.4)
West	8,330 (27.6)	5 (13.2)
Puerto Rico	207 (0.7)	0 (—)
**Experiencing homelessness**	**NA**	**11 (40.7)**
Yes	NA	5 (45.5)
No	NA	6 (54.5)
Unknown	30,183	16
**Clinical**		
**Sexual or intimate contact in the 3 wks before symptom onset**	**18,385 (60.9)**	**16 (42.1)**
Sexual contact in the past 3 wks	15,061 (81.9)	10 (62.5)
Recent partners exclusively men	12,759 (69.4)	9 (56.2)
Recent partners women or other genders (no men)	898 (4.9)	0 (—)
Recent partners include men and other genders	503 (2.7)	0 (—)
Gender of all partners unknown/Not specified	901 (4.9)	1 (6.3)
No recent sexual contact	3,324 (18.1)	6 (37.5)
Unknown	11,798	22
Interval from illness onset to testing, days, median (IQR)**	**7 (4–10)**	**7 (3–10)**
**HIV-positive or immunocompromised^††^**	**13,549 (44.9)**	**33 (86.8)**
Yes, HIV-positive	5,186 (38.3)	31 (93.9)
Yes, other immunocompromising conditions	654 (4.8)	2 (9.1)
No	7,709 (56.9)	0 (—)
Unknown	16,634	5
**Received JYNNEOS vaccine** ^§§^	**11,316 (37.5)**	**13 (34.2)**
Yes	8,238 (72.8)	1 (7.7)
No	3,078 (27.2)	12 (92.3)
Unknown	18,867	25

**Abbreviation:** NA = not available.

* Mpox was listed as directly causing or significantly contributing to death on the death certificate.

**^†^** Percentages were calculated with nonmissing data.

^§^ Among decedents whose death was mpox-associated (38), self-reported data on gender identity was available for 28 (73.7%) decedents. Sex assigned at birth was substituted for 10 (26.3%) decedents for whom gender identity was missing.

^¶^ https://www2.census.gov/geo/pdfs/maps-data/maps/reference/us_regdiv.pdf (Accessed March 24, 2023). Puerto Rico is not included in any U.S. Census Bureau region.

** Illness onset is the date any reported symptoms first started.

**^††^** Non-HIV immunocompromising conditions included renal transplant (one) and uncontrolled diabetes (one).

^§§^ At least 1 dose.

Among 27 (71.0%) decedents with available clinical data, the median interval from symptom onset to death was 68 days (range = 1–146 days; IQR = 50–86 days), and 87.0% (20 of 23) were admitted to an intensive care unit during hospitalization (Table 2). Overall, 25 (92.6%) of 27 decedents with available treatment information received mpox-directed medical therapeutics^§§§^ at some point during their clinical care, including 25 who received tecovirimat, 18 of 24 who received vaccinia immunoglobulin intravenous (VIGIV), nine of 22 who received cidofovir, and six of 15 who received brincidofovir. Among the 25 decedents who received tecovirimat, 15 (60.0%) received it within 3 days of mpox diagnosis; however, for six (24.0%) decedents, tecovirimat was not administered until ≥3 weeks after diagnosis (median overall interval = 2 days; IQR = 0–20 days). Among 24 decedents with available data, all had lesions described as necrotic, diffuse, or worsened after a 14-day course of tecovirimat.

**TABLE 2:** Selected clinical characteristics* of mpox-associated deaths with available clinical data (N = 27) — United States, May 10, 2022–March 7, 2023

Characteristic (no. with information)	Mpox-associated deaths, no. (%)^†^
**Clinical**	
**Days from symptom onset to death (26), median (IQR)**	**68 (50–86)**
**Admitted to ICU**	**23 (85.2)**
Yes	20 (87.0)
No	3 (13.0)
Unknown	4
**Medical treatment^§^**	
**Received any mpox-directed treatment**	**27 (100.0)**
Yes	25 (92.6)
No	2 (7.4)
Unknown	0
**Days from first mpox treatment date to death (21), median (IQR)**	**58 (32–66)**
**Specific mpox-directed treatment received**	
**Tecovirimat**	**27 (100.0)**
Yes	25 (92.6)
No	2 (7.4)
Unknown	0
**Interval from mpox diagnosis to initiation of tecovirimat treatment (20), days, median (IQR)**	**2 (0–20)**
**VIGIV**	**24 (88.9)**
Yes	18 (75.0)
No	6 (25.0)
Unknown	3
**Cidofovir**	**22 (81.5)**
Yes	9 (40.9)
No	13 (59.1)
Unknown	5
**Brincidofovir**	**15 (55.6)**
Yes	6 (40.0)
No	9 (60.0)
Unknown	12
**Necrotic, diffuse, or worsened lesions^¶^**	**24 (88.9)**
Yes	24 (100.0)
No	0 (—)
Unknown	3
**Received steroids for mpox complications or IRIS concerns**	**24 (88.9)**
Yes	13 (54.2)
No	11 (45.8)
Unknown	3
HIV-positive or immunocompromised**	**27 (100.0)**
HIV-positive	25 (92.6)
CD4 ≥500	0 (—)
CD4 ≥200 to <500	0 (—)
CD4 ≥50 to <200	1 (4.2)
CD4 <50	23 (95.8)
CD4 Unknown	1
Immunocompromised (HIV-negative)	2 (7.4)
Unknown	0
**Receiving ART (HIV-positive persons)**	**22 (88.0)**
Yes, before mpox diagnosis	2 (9.1)
Yes, after mpox diagnosis	19 (86.4)
No, refused	1 (4.5)
Unknown	3
**Interval from mpox diagnosis to initiation of ART (9), days, median (IQR)**	** 15 (5–26)**

**Abbreviations**: ART = antiretroviral treatment; ICU = intensive care unit; IRIS = immune reconstitution inflammatory syndrome; VIGIV = vaccinia immune globulin intravenous.

* Reported or collected during clinical consult with treating physician.

**^†^** Percentages calculated with nonmissing data. Percentages calculated based on total number in each subgroup.

^§^ Treatment classification includes report of tecovirimat (oral or intravenous), VIGIV, cidofovir, or brincidofovir in the clinical consultation data.

^¶^ Reported during clinical consultation with treating physician or through expert assessment of photographs.

** Non-HIV immunocompromising conditions included renal transplant (one) and uncontrolled diabetes (one).

Two decedents did not receive any mpox therapeutics based on clinician discretion; in one case because of concerns regarding contraindication associated with other comorbidities. The other decedent was on ART for HIV with an undetectable viral load at the time of initial assessment for mpox care. Approximately 1 month later, at a wellness check, the patient was found deceased with diffuse lesions characteristic of mpox. Seven of 27 decedents for whom information was available refused therapeutic or intravenous medications or left the hospital against medical advice at some point during their clinical course of treatment. Among 24 decedents with information on receipt of steroids, more than one half (54.2%) received steroids for mpox complications or concerns about immune reconstitution inflammatory syndrome (IRIS), a hyperinflammatory response that can occur in patients with HIV during the first 6 months of ART (*6*).

Nearly all decedents with complete data on HIV infection were HIV-positive (93.9%; 31 of 33). Among 24 decedents with HIV and available data, all (100%) had CD4 counts <200 cells/mm^3^; 23 (95.8%) had CD4 counts <50 cells/mm^3^. Among the two immunocompromised decedents who did not have HIV, one was presumed to have been immunocompromised as a consequence of undiagnosed diabetes; the patient experienced diabetic ketoacidosis, a severe life-threatening complication of diabetes, at the time of mpox diagnosis. The second decedent was severely immunocompromised because of a recent renal transplant complicated by acute rejection.

Only two of the 25 persons with HIV (8.0%) reported taking ART before mpox diagnosis; in one of these decedents, HIV was poorly controlled. ART was initiated for 19 of the 20 decedents who were not receiving ART, including one decedent who received a diagnosis of HIV infection 5 days after the mpox diagnosis. One decedent refused treatment for advanced HIV. ART treatment status was not known for three of the 25 decedents with HIV. ART was either delayed or interrupted for seven decedents because of clinician concerns about IRIS.

## Discussion

During the 2022 mpox outbreak, 1.3 mpox-associated deaths per 1,000 cases occurred in the United States and approximately 1.2 deaths per 1,000 mpox cases occurred worldwide.^¶¶¶^ Nearly all U.S. mpox decedents were immunocompromised at the time of diagnosis. Almost 90% of U.S. mpox-associated deaths occurred in Black men, whereas fewer than one in three mpox survivors were Black men. Although mpox exposure data were not available for all decedents, nearly all cisgender male decedents who reported recent sexual contact reported cisgender male partners.

Most decedents received one or more prompt mpox-directed treatments and intensive care. However, nearly one quarter of decedents experienced delays of 3–7 weeks between diagnosis and treatment, and two patients did not receive any mpox-directed treatment. Before the 2022 mpox epidemic, severe cases mainly occurred in resource-constrained settings where transmission is endemic and where treatments, such as tecovirimat, cidofovir, and VIGIV, are not routinely available (*6*).

The lengthy course of illness experienced by most decedents is likely related to a reduced capacity to respond to infection because of co-occurring immunocompromise. When mpox is suspected, providers should consider early treatment with mpox-directed therapy, especially for patients with immunocompromising conditions who are at highest risk for severe outcomes.

The gender and racial disparities in mpox-associated deaths align with previous reports, in which most patients hospitalized for severe manifestations of mpox were Black men with uncontrolled HIV (*4*) and parallel racial and ethnic disparities in HIV infection and mortality. In 2020, 75% of all-cause deaths among adults with HIV occurred in males, 39% of whom were Black males.**** Disparities and barriers are apparent at all levels of HIV care including recognition of HIV risk, access to testing, and access to and receipt of preexposure prophylaxis and ART (*7*).

Most decedents with advanced HIV were not on ART at the time mpox was diagnosed. Boosting immune function via ART and avoidance of immunosuppressives, such as steroids, are critical to mpox recovery (*6*). ART was delayed or discontinued for several decedents because of concerns of IRIS, wherein a sudden improvement of immune function paradoxically worsens an infection. To date, no evidence exists indicating that IRIS contributes to poor mpox outcomes. 

The Ending the HIV Epidemic initiative^††††^ has highlighted the need to address HIV transmission in the United States through response, prevention, diagnosis, and treatment efforts. All patients with suspected mpox should be evaluated for preexisting immunocompromising conditions such as HIV (*7*). Persons with diagnosed mpox who have HIV (even if newly diagnosed) who are not on ART should be started on ART as soon as possible. For persons who are HIV-negative, indications for HIV pre-exposure prophylaxis should be assessed. 

Previous studies of mpox patients, in Nigeria, have documented anxiety, depression (*8*), and suicide (*9*). It has been suggested (*9*) that these outcomes resulted from stigma and the implications of acquiring a sexually associated infection, such as mpox, as well as the isolation experienced during prolonged treatment. Black and Hispanic or Latino males in the United States might face additional psychosocial pressures related to language barriers, homophobia, and discrimination (*10*). One suicide was noted among the decedents of non–mpox-associated deaths, and one decedent persistently declined treatment for HIV. These data highlight the need to strengthen psychosocial support services as part of the mpox epidemic response.

The findings in this report are subject to at least two limitations. First, deaths might be undercounted. Delays in reporting laboratory results, cases, and deaths are expected because reporting guidance was developed concurrently with the outbreak. Because reports from clinical consults were passively obtained, some deaths related to patients with mpox might not have been recorded. Second, some data (e.g., housing status, treatment, HIV status, and CD4 count) were more frequently available for decedents than for survivors because of additional reporting requirements for those who received intensive mpox treatments, thus limiting the comparisons between these two groups.

These findings highlight the importance of integrating prevention, testing, and treatment for multiple sexually associated infections (e.g., mpox and HIV, among others). Equitable access to prevention, treatment, and engagement and retention in care for both mpox and HIV should be prioritized, particularly among Black men and other persons at risk for sexually associated infections. These results underscore previous recommendations that providers offer HIV testing to all patients with probable or confirmed mpox and consider early mpox-directed treatment in highly immunocompromised patients (*4**,**5**,**7*). Further, combining therapies for mpox and boosting immune function might also reduce mortality from severe mpox (*5*).

**Summary**

**What is already known about this topic?**

Severe manifestations of mpox have occurred in the United States, particularly among persons with uncontrolled viral spread resulting from moderately to severely immunocompromising conditions.

**What is added by this report?**

Thirty-eight mpox-associated deaths occurred in the United States during May 10, 2022–March 7, 2023 (1.3 mpox-associated deaths per 1,000 cases). Most decedents were non-Hispanic Black or African American (87%) persons and cisgender men (95%). Among 24 decedents with HIV for whom data were available, all had advanced HIV, typically with a CD4 count <50.

**What are the implications for public health practice?**

Equitable and early access to prevention and treatment for both mpox and HIV is critical to reducing mpox-related mortality.

## Footnotes

* These authors contributed equally to this report.

† https://www.cdc.gov/poxvirus/mpox/response/2022/us-map.html (Accessed March 20, 2023).

§ Among mpox-associated deaths (38), data on gender identity was available for 28 (73.7%) decedents. For 10 (26.3%) persons for whom self-reported gender was missing, sex assigned at birth was substituted for gender identity.

¶ Case counts include those who received a positive test result for either *Monkeypox virus *or orthopoxvirus.

** https://www.cdc.gov/nndss/index.html (Accessed March 20, 2023).

†† CDC provides clinical consultations upon request to jurisdictions and clinicians treating patients with mpox. Health care providers seeking additional clinical guidance can contact the CDC Emergency Operations Center by telephone (770-488-7100) or by email (eocevent482@cdc.gov).

§§ Discrepancies between laboratory reports and the cause of death as reported on the death certificate prompted three jurisdictions to seek clarification to reconcile the data.

¶¶ 45 C.F.R. part 46.102(l)(2), 21 C.F.R. part 56; 42 U.S.C. Sect. 241(d); 5 U.S.C. Sect. 552a; 44 U.S.C. Sect. 3501 et seq.

*** Causes of death included suicide and drug overdose.

†††
https://www2.census.gov/geo/pdfs/maps-data/maps/reference/us_regdiv.pdf

§§§ Treatment classification includes report of tecovirimat (oral or intravenous), VIGIV, cidofovir, or brincidofovir in the clinical consultation data.

¶¶¶ https://www.cdc.gov/poxvirus/mpox/response/2022/world-map.html (Accessed March 5, 2023).

**** The number of deaths from all causes in patients with HIV infection was calculated using CDC’s Atlas Plus. https://gis.cdc.gov/grasp/nchhstpatlas/tables.html

†††† https://www.cdc.gov/endhiv/index.html
