# *Erratum: *Vol. 72, No. 15

**DOI:** 10.15585/mmwr.mm7231a5

**Published:** 2023-08-04

The report, “Epidemiologic and Clinical Features of Mpox-Associated Deaths — United States, May 10, 2022–March 7, 2023,” contained several errors.

On page 407, in Table 1, the eleventh row heading should have read, “Received **smallpox** vaccine^§§^,” and under the column “Decedents (n = 38),” the number and percentage for the row listed under “Yes” should have read **1 (7.7)^¶¶^**. On page 407, under the footnotes for Table 1, the seventh footnote, denoted by the double section sign (^§§^), should have read, **“^§§^** At least 1 dose **of smallpox or JYNNEOS vaccine. Among some vaccine recipients, it is difficult to distinguish when vaccine was received and whether indication for use was prevention of smallpox or mpox.”** A newly added eighth footnote, denoted by the double paragraph symbol (^¶¶^), should have read, **“^¶¶^ One person who died reported receiving smallpox vaccination in childhood, even though this decedent was aged <50 years (routine smallpox vaccination ended in the United States in 1972).” **

On page 409, the list of acknowledgments should have read, “Health care providers caring for mpox patients; public health responders from U.S. state and local departments of health; Isaac Ghinai, Janna L. Kerins, Massimo Pacilli, Hillary Spencer, Irina Tabidze, Chicago Department of Health; Rafael M. Mendoza, Jenniffer Rivas, Florida Department of Health in Broward County; Kristine Aviles, Florida Department of Health in Hillsborough County; Elizabeth Feinstone, Kristy Flom, Florida Department of Health in Osceola County; Madison Asbell, Cyndy Fohrman, Brenda Parduhn, Kira Richardson, Indiana Department of Health; Darby McDermott, Mojisola Ojo, Alex X. Zhang, New Jersey Department of Health; Courtney Dewart, CDC and Ohio Department of Health; Kara Tarter, Ohio Department of Health; **Haley Blake, Southern Nevada Health District**; Kevin Morris, Caleb Wiedeman, Tennessee Department of Health; Rania Milleron, Texas Department of State Health Services; Laura Bachmann, Adelaide Balenger, Denisse Descamps, Romeo R. Galang, Kaylea Nemechek, Suzanne Newton, Sheila Roy, Ruth Stefanos, CDC; CDC Clinical Escalations Team and health department personnel who consulted CDC for complicated cases of mpox; CDC clinical officers who perform consultations.”

